# “All‐on‐4” and “All‐on‐6” treatment concept applied using computer‐guided surgery in a patient: Case report with a 2‐year follow‐up

**DOI:** 10.1002/ccr3.7101

**Published:** 2023-03-14

**Authors:** Ahmed Heji Albaqawi

**Affiliations:** ^1^ Department of Restorative Dental Science College of Dentistry University of Haˈil Haˈil Kingdom of Saudi Arabia

**Keywords:** edentulous jaw, immediate implant, immediate rehabilitation

## Abstract

This case aimed to assess the clinical and radiographic outcomes of the All‐on‐4 and the All‐on‐6 concept using three‐dimensional computer‐assisted treatment planning, and computer‐guided surgery. Two years after the treatment, the “All‐on‐4” and “All‐on‐6” concepts provided effective treatment for immediate restoration and showed predictable outcomes in a completely edentulous patient.

## 1 INTRODUCTION

In edentulous patients, complete dentures are considered the most appropriate treatment option for maintaining normal speech and an aesthetically pleasing appearance and facilitating adequate mastication of food.^1^ A number of approaches have been developed to restore edentulous upper and lower jaws.^2^

Primary stability refers to the initial mechanical anchorage of the implant to the bone. It is influenced by numerous factors, such as bone quantity and quality, the geometric design of the implant, surgical technique, and insertion torque. The secondary (or biological) stability is provided by gradual bone remodeling on the implant surface in the empty chambers among threads during the first 2 weeks, either by new bone formation or by interfacial remodeling of pre‐existing bone, depending on the contact between the implant body and the bone tissue.^3^, ^4^

Case studies were performed in the 1990s to record the immediate loading procedures. When compared to the traditional two‐stage strategy, the first data showed a higher risk of implant loss. Clinicians responded by inserting up to 13 implants in one jaw in some cases.^5^, ^6^ In 1999, however, Brånemark et al.^7^ proposed the Novum® method, which included a prefabricated surgical guide and mandibular prosthetic parts. This allowed for the loading and delivery of a final fixed restoration on only three implants. While groundbreaking at the time, the technique was deemed too complex, as it addressed only a small number of patients with excellent bone quality.

In 2003, the “All‐on‐4” treatment concept was introduced for prosthetic rehabilitation based on only four implants: two in the anterior region of the jaw, which are oriented straight, and two in the posterior region, which are tilted distally.^8^ Denture stability is enhanced with longer implants in the bone.^9^ An immediately functional provisional fixed full‐arch prosthesis can be loaded on the day of surgery. With this concept, bone transplantations are avoided; surgical time and costs are reduced.^10^, ^11^ Moreover, high survival rates of fixed dentures were found, for example, 99.2% in a study with a 10‐year follow‐up.^12^ “All‐on‐4” treatment concept was studied extensively by many investigators.^9^, ^13^, ^14^, ^15^, ^16^, ^17^, ^18^, ^19^, ^20^, ^21^, ^22^, ^23^

This case report aimed to assess the clinical and radiographic outcomes of the “All‐on‐4” treatment concept in the mandible and the “All‐on‐6” concept in the maxilla in a completely edentulous patient with severe bone atrophy in the maxilla using three‐dimensional (3D) computer‐assisted treatment planning, and computer‐guided surgery. The “All‐on‐4” and “All‐on‐6” concepts are applied in this case to use most of the remaining bone in atrophic jaws, enabling immediate function and avoiding bone augmentation that increase treatment costs and risks for patients.

## 2 CASE PRESENTATION

*Treatment indication*: A 62‐year‐old male patient presented to the Department of Prosthetic Dentistry at the University of Freiburg. The patient's main complaint was an unpleasant appearance and inability to chew. The patient wishes to have a fixed work prosthesis and improve the aesthetic of the teeth. The patient has no medical problems and he is not taking any medication. Also, the patient was a non‐smoker. The previous socioeconomic background of the patient was low income and he has a high‐stress job. Furthermore, he lacks access to health care. All of these could lead to the loss of his teeth and cause dental carious. Clinical examination revealed maxillary and mandibular partial edentulism with remaining hopeless teeth. The patient has no TMJ or muscle problems. The patient's profile reveals a decrease in the low vertical dimension and loss of the lip's support. Both the mandible and maxilla were affected by significant horizontal bone resorption with partial loss of the vertical dimensions (Class III alveolar crestal defects according to Seibert). Given the advanced bone atrophy, the “All‐on‐4” and “All‐on‐6” treatment concepts were proposed to the patient as the most suitable treatment option.

The treatment options were (i) conventional complete full dentures, (ii) overdenture on dental implants with ball‐head attachments, (iii) “All‐on‐4” and “All‐on‐6” treatment concept with the fixed dental prosthesis.

*Surgical and prosthetic procedures*: All remaining teeth were extracted before 4 months of implant placement, and the patient was rehabilitated with an immediate complete denture.

Preliminary impressions were made using an irreversible hydrocolloid impression material (Pluralgin Super®, Pluradent AG & Co KG). After preparing the primary casts, mandibular and maxillary trays were fabricated using a light‐cured resin (Palatray® LC, Heraeus‐Kulzer, D‐Hanau). A thin layer of zinc oxide eugenol (ZnOE) paste was applied to the maxilla (Kelly®, Ubert) and to the mandible (SS White®, Ubert). Determination of the vertical relation, the centric relation record, try‐in of complete dentures, and completion of the dentures were performed before the digital volume tomography (DVT). Also, the bone dimension for implants placements was assessed using 3D virtual planning.

**FIGURE 1:**
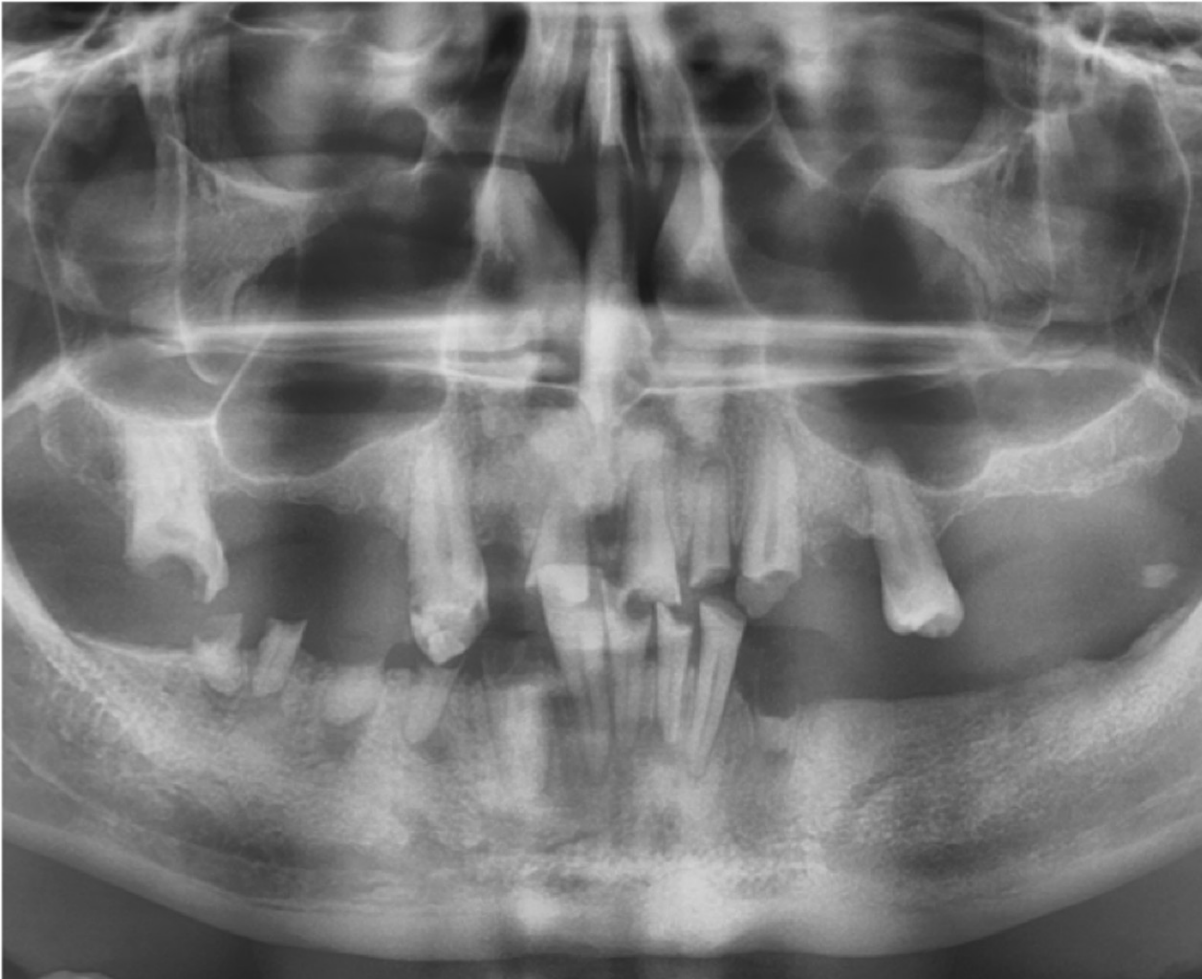
Preoperative radiograph of the treated case.

**FIGURE 2:**
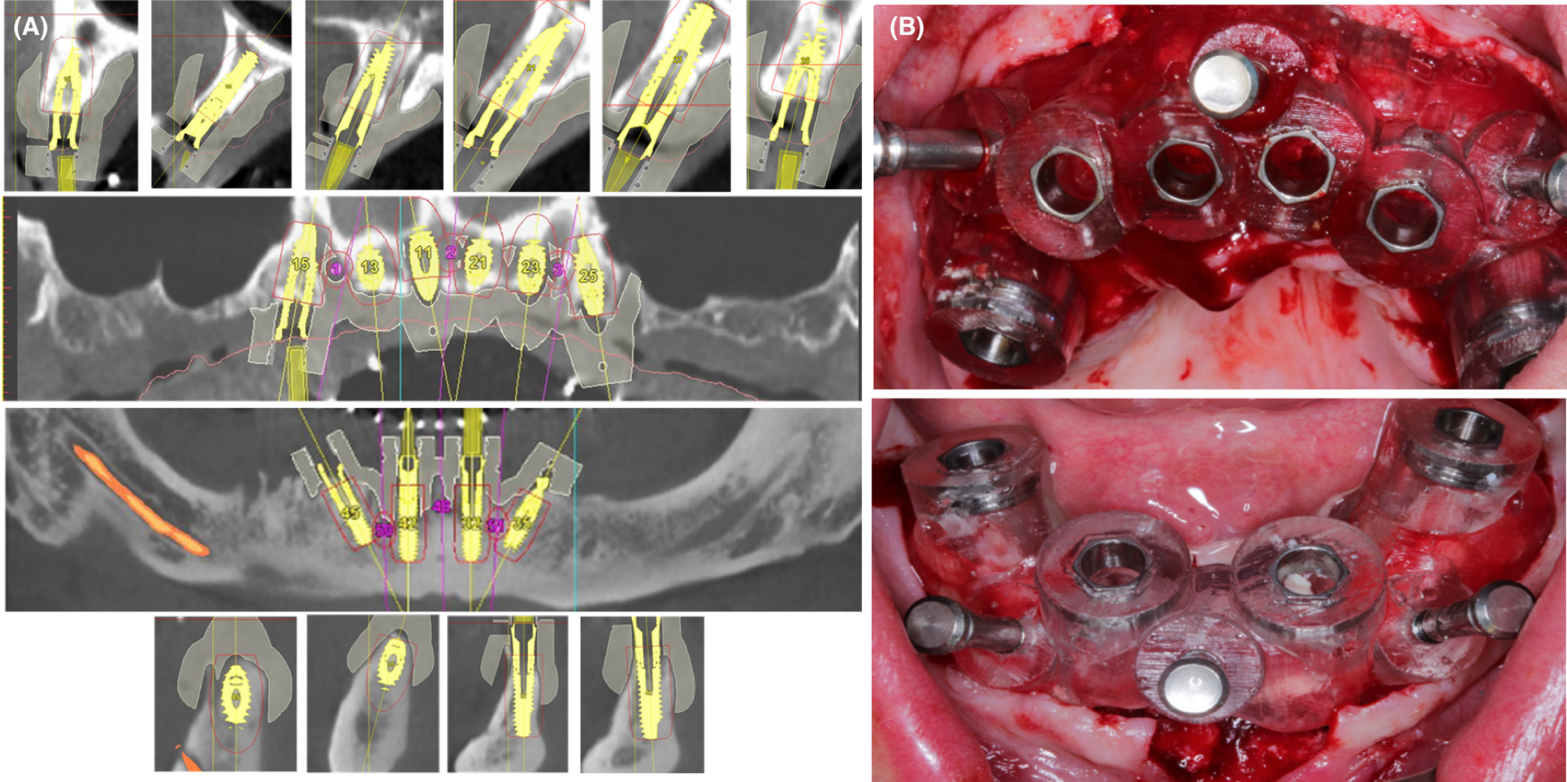
(A) Digital volume tomography before implant placement, (B) Bone‐supported surgical guide during the surgical procedure.

Preoperative imaging was employed to obtain a two‐dimensional image of the bone structures, as shown in (Figure 1). One of the most important steps during DVT was that the patient was wearing the denture, with each tooth in the denture attached to the gutta‐percha to show the long axis of the tooth. For implant planning, the computer software SimPlant® (Materialize Dental NV) was used for analysis of DVT data (Figure 2A). After 3D virtual planning, the data for the optimal position and inclination of the implants were sent via SimPlant® software to the production center, which fabricated template surgical‐precision resin cylindrical guides with titanium holes.

One hour before the prosthetic surgery, the patient received antibiotic prophylaxis (amoxicillin, GlaxoSmithKline, total dose of 2 g, tablets for oral administration) and rinsed his mouth using a mouthwash (chlorhexidine digluconate 0.2%, Corsodyl, GlaxoSmithKline). Sedation with local anesthesia was used (four carpules with epinephrine at a 1:100,000 concentration).

The classic surgical procedure was used in both jaws. During surgery, six implants (Xive S plus, Ø3.8 mm × 13 mm length, Dentsply Sirona) were placed in the basal bone of the upper jaw (in the regions 15, 13, 11, 21, 23, 25), and four implants (Xive S plus, Ø3.8 mm × 13 mm length, Dentsply Sirona) were inserted in the basal bone of the lower jaw (32, 35, 42, 45) with the help of bone‐supported surgical guide was fixed with three anchor pins to the basal bone. Four implants (15, 25, 35, and 45) were inserted and tilted at an angle of 30° to the bone, while all other implants were oriented straight. All implants were torqued to 30–50 newton centimeters (Ncm) to allow immediate rehabilitation (Figure 2B).

The Xive S Plus screw implants were made of commercially pure titanium grade II was used in this study. The surface of implants consisted of horizontal threads and a sand‐blasted/acid‐etched micro‐structure which results in a micro‐roughness of >2 μm. The Xive MP abutments angled at 30° (Dentsply Friadent) were connected to implants 15, 25, 35, and 45 as the baseline pictures. The Xive MP straight abutment was connected to all other implants to make all abutments parallel to each other. All abutments were torqued to 30 Ncm. The SmartFix® concept (Dentsply Sirona) was used during the operation to ensure parallelism of the Xive MP abutment, which helps the fit with immediately relining the provisional prosthesis.

**FIGURE 3:**
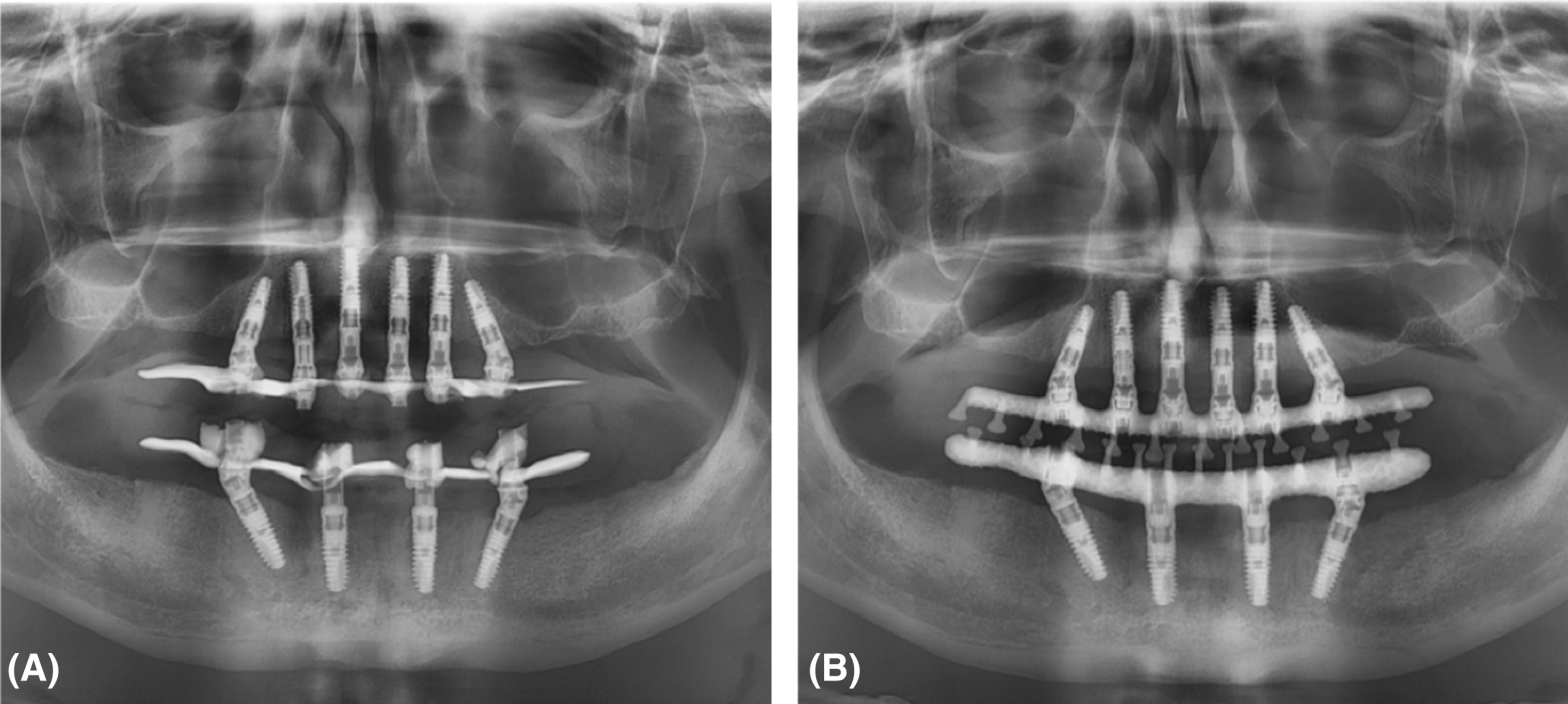
(A) Provisional prosthesis with thin metal framework, (B) Final prosthesis with milled titanium frameworks.

An acrylic, screw‐retained provisional prosthesis with thin metal framework was loaded immediately 3 h after surgery due to relining the denture (Figure 3A). The patient received postoperative amoxicillin (500 mg tablets, three times per day) for 5 days. In addition, he was given oral hygiene instructions. Sutures were removed at the 2‐week follow‐up appointment.

**FIGURE 4:**
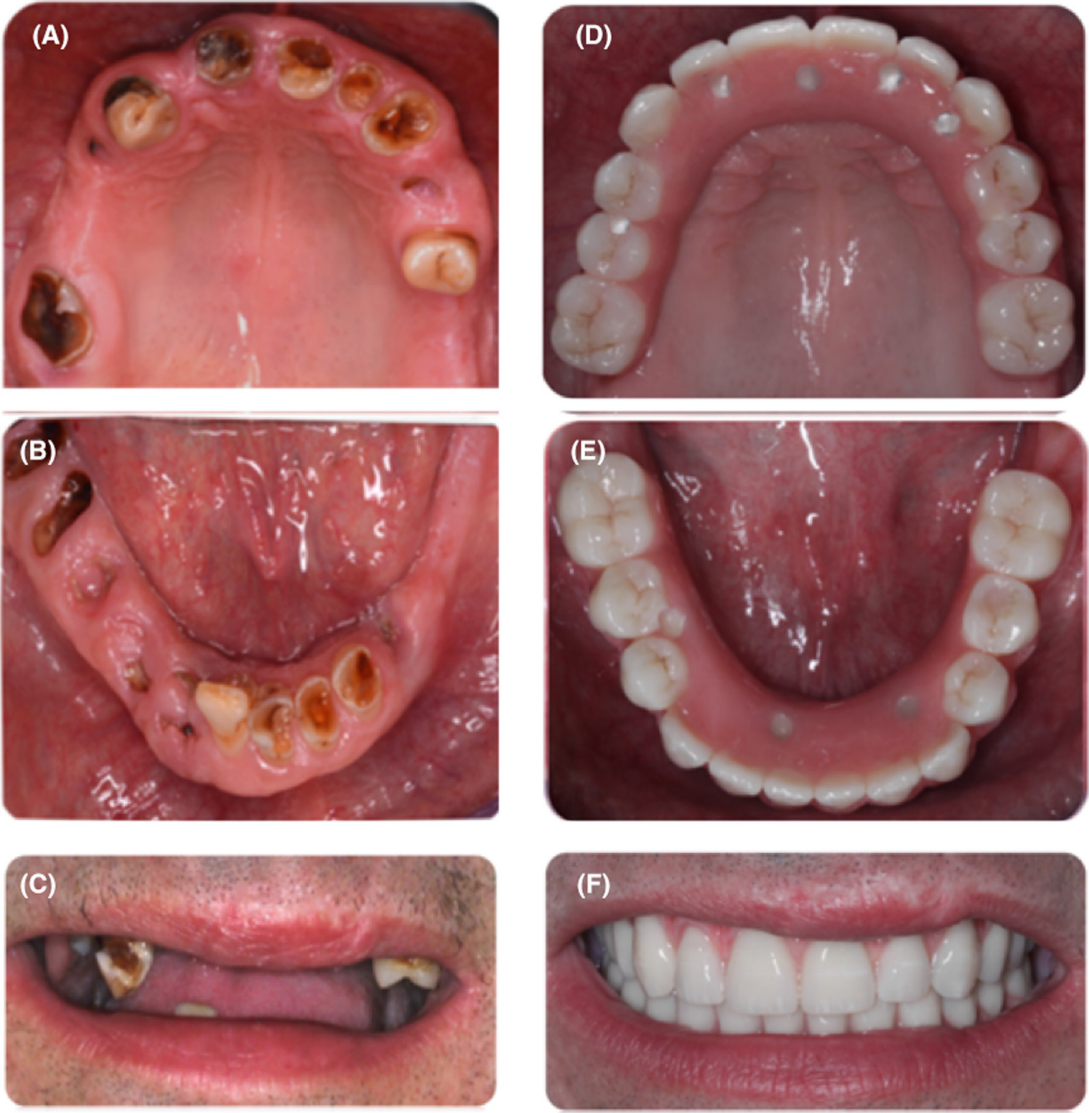
(A–C) Preoperative clinical view of the treated patient, and (D–F) after prosthesis insertion.

After 1 year, the radiographic examination of the acrylic, screw‐retained provisional prosthesis with thin metal framework with dental implants was showed, there is no biological complication (Figure 3A). The definitive computer‐aided design/computer‐aided manufacturing (CAD/CAM)‐milled titanium frameworks were fabricated and tried intraorally for fine adjustments. The definitive denture base was made of light‐cured acrylic (Palatray® LC, Heraeus‐Kulzer), and the appropriate artificial teeth were selected on the basis of the size, shape, color/shade, and naturalness of the teeth. (Integral®, Merz AG). The final prosthesis was screw‐retained. The patient was instructed regarding proper prosthetic care. Radiographic examination at the follow‐up appointment 2 years after surgery revealed good bone healing and no peri‐implant complications (Figure 3B). The patient was satisfied with the aesthetic, phonetic, and functional outcomes of the prosthesis (Figure 4).

*Follow‐up and maintenance*: The patient was given instructions on how to practice oral hygiene procedures on a daily basis, including the use of an interdental brush and super floss in addition to the traditional brush and floss. Every 3 months for the first year and then every 6 months after that, the patient was invited to come in for routine follow‐ups. One week after immediate loading, the patient was scheduled for the first control appointment. Further follow‐up visits were planned for 3 months, 6 months, 1 year, and 2 years. The patient was given detailed post loading instructions, including how to use dental floss and an interdental brush. During the maintenance period, the patient received periodontal care as required for both provisional and definitive prosthesis.

The following summarizes the treatment algorithm of this case:

Step 1: Case selection and treatment planning.

Step 2: Extraction of remaining teeth and healing period.

Step 3: Insertion of immediate complete dentures.

Step 4: DVT for implant planning placement.

Step 5: Implants placement with fabricated template surgical‐precision resin cylindrical guides and provisional prosthesis with thin metal framework.

Step 6: Final prosthesis with (CAD/CAM)‐milled titanium frameworks.

Step 7: Follow‐up and maintenance protocol.

## 3 DISCUSSION

In situations when the implant is intended to be placed close to a vital anatomical structure (next to the inferior dental nerve, the maxillary sinus, etc.), computer‐guided surgery may be more appropriate.^24^ Due to its high level of precision, fully‐guided surgery is the most appropriate procedure in certain situations within guided surgery. Guided surgery has limits, such as higher costs, the requirement for suitable anatomical conditions in terms of buccal opening, and adequate adjustment of the surgical guides, even though the accuracy gained with guided surgery is higher than with freehand surgery.^25^

Given the highly atrophic clinical condition of the case described here, combinations of six implants in the upper jaw and four implants in the lower jaw were considered the most advisable approach. By placing more implants in the maxilla, the survival rate of the maxillary prosthesis was expected to be very high and similar to that of the mandibular prosthesis. The maxilla is known to generally withstand lower mechanical forces than the mandible due to its relatively thin cortical layer and low density of spongiosa.^26^ Thus, when the “All‐on‐4” concept is applied to both jaws, the failure rate is usually significantly higher for maxillary implants than mandibular implants.^27^ The number of implants recommended (according to the S3 guideline “DGZMK”) in the upper jaw with fixed restoration is six implants.^28^ Therefore, in the present study, six implants were placed in the maxilla; however, in the mandible, only four implants were inserted.

Previous research has demonstrated that computer‐guided surgery is more accurate than nonguided or freehand surgery in terms of implant placement.^29^, ^30^ Jung et al.^31^ conducted a comprehensive study on accuracy based on studies of computer‐guided surgery for dental implant insertion. In general, the accuracy was higher in in vitro and ex vivo models than in in vivo experiments. In this regard, these authors offered features such as improved access, vision, and control of the osteotomy axis, the lack of patient movements, and the absence of saliva and bleeding to explain why higher deviations were detected in clinical tests in vivo. Bover‐Ramos et al.^32^ conducted a comprehensive review and meta‐analysis to assess the accuracy of implant placement utilizing computer‐guided surgery and to compare virtual planning and outcomes by research type (in vitro, cadaver, or clinical). When compared to in vitro research, clinical and cadaver investigations had worse implant placement accuracy, particularly in terms of apical and angular deviations. Katherine Turbush and Ilser Turkyilmaz.^33^ investigated the accuracy of three types of stereolithographic surgical guides in implant placement. They discovered that using a stereolithographic bone‐surgical guide resulted in reduced deviation. As a result, the use of computer‐guided surgery has been limited to the surgical benefits of implant therapy. Prosthetic therapy must still be performed in accordance with standard guidelines. However, the link used to convey prosthetic information to the patient is critical, and precise reference points are necessary to place the implants so that prefabricated prosthetics fit precisely.^34^ Formalized paraphrase as a result, in this case study, a stereolithographic bone‐surgical guide was employed to reduce deviation, and the prosthetic framework was created using CAD CAM to ensure a precise fit. Individualized computer‐assisted planning and computer‐guided surgery may be used in cases of extensive bone atrophy to optimally exploit the remnant alveolar ridge and denser bone areas for implant location and inclination. As a result, surgical invasiveness was reduced, while the primary stability of the prosthesis was increased. Furthermore, a recent systematic study found that implant failure rates in the free‐hand implant placement category were nearly three times greater than those in the guided implant placement category.^35^ Furthermore, this approach is still employed in many contemporary clinical cases.^14^, ^15^

The length of all implants exceeded 10 mm, a length commonly associated with a better survival rate.^36^ Furthermore, the presence of critical anatomical features of the peri‐maxillary and mandibular implants, as well as moderate to severe bone resorption, were the primary grounds for defining implant length, according to two studies.^37^, ^38^ Therefore, the length of the implants in the present study was 13 mm according to the analysis of DVT data via 3D software virtual planning and the specified angles of the MP abutments. With the use of DVT, it is possible to assess not only the bone density but also the anticipated bone contact with the implant, differentiating between areas of varying quality and thickness. This makes it possible to anticipate primary stability during preoperative diagnosis. It enables case‐by‐case selection and ideal implant location.^39^

Regarding distal implant placement, many authors have reported that distal implants are placed with varying angulations of 25°, 30°, and 45° between the implants and the prosthetic abutment inclination,^8^, ^40^, ^41^ and these degrees of inclination are primarily determined by the quality of the bone in terms of length‐width and anatomical location.^12^, ^42^ Furthermore, a finite element study revealed that the stress distribution on peri‐implant cortical bone increased with an increasing inclination angle and decreased the number of implants.^43^ Other investigations, however, have found no difference in stress on cortical bone between angled and nonangled implants on a prosthetic superstructure utilizing splinted and nonsplinted implants.^44^ In this study, the inclination angle was selected utilizing analysis jaw models on 3D virtual implants planning with angled abutments, as well as SmartFix® (Dentsply Sirona) during the procedure. As a result, the implants in the mandible were positioned at 15° in the current investigation.

For the definitive prosthesis, a framework reinforced with computer numeric controlled milled titanium was chosen, as breakage of prostheses or the metal base of the prosthesis were reported to be less frequent, and the framework fit was found to be better than that of conventional frameworks.^45^

Peri‐implantitis has been observed in some patients after an average follow‐up period of 1–2 years.^46^ In our case, however, there were no signs of biological complications around the implants at 2 years after surgery. Generally, at all appointments, the patient was instructed and advised to maintain adequate mouth oral hygiene. However, given that an increasing number of patients retain their implants for a long period of time (>10 years), it is possible that some infections around implants develop slowly and that, over time, peri‐implantitis will become a frequent consequence to implant therapy.

Some of the limitations of the present study are related to the “All‐on‐4” and “All‐on‐6” treatment concepts in combination with computer‐guided surgery represents a sensitive technique that requires the skills and experience of a well‐trained implant surgeon to avoid treatment failures, such as implant loss or prosthetic fractures.^27^ The advantages of this treatment concept, however, justify and far outweigh any efforts in managing the learning curve. This concept requires the presence of at least 10 mm bone thickness. It requires complex and precise lab fabrication methods. It is also an expensive type of treatment.

## 4 CONCLUSION

The “All‐on‐4” and “All‐on‐6” concepts provided effective treatment for an immediate restoration. This study showed that “LL‐on‐4” and “All‐on‐6” concepts offered predictable outcomes in a completely edentulous patient.

### CLINICAL SIGNIFICANCE

The combination of the “All‐on‐4” and “All‐on‐6” treatments with computer‐assisted planning can maximize the use of available bone for optimum implant anchorage and immediate loading of the provisional prosthesis.

## AUTHOR CONTRIBUTIONS

A.H.A. contributed to surgical and prosthetic procedures, writing the original draft and reviewing and editing the final manuscript. The author revised and approved the final paper.

## CONFLICT OF INTEREST STATEMENT

The author declares no conflict of interest.

## CONSENT

Written informed consent was obtained from the patient to publish this case report in accordance with the journal's patient consent policy. The patient provided written informed consent for surgical prosthetic procedures to publish this case report, including a description of this case, medical and dental information, and images.

## Data Availability

The data that support the findings of this case report study are available on reasonable request from the author. The patient agreed to participate in the present investigation. He provided an information sheet and sign a written informed consent form.
